# I can’t feel your face: callous-unemotional traits, social anxiety, and approach-avoidance behaviour in conduct disorder

**DOI:** 10.1186/s13034-024-00831-y

**Published:** 2024-11-27

**Authors:** Laura M. Derks, Eni S. Becker, Mike Rinck, Martin Holtmann, Tanja Legenbauer, Wolf-Gero Lange

**Affiliations:** 1 Department for Child and Adolescent Psychiatry, Psychosomatic and Psychotherapy, LWL University Hospital of the Ruhr-University Bochum, Hamm, Germany; 2 Behavioural Science Institute, Radboud-University Nijmegen, 500 HB, Nijmegen, The Netherlands

**Keywords:** Conduct disorder, Callous-unemotional traits, Approach- and avoidance behaviour, Social anxiety, Interpersonal distance

## Abstract

**Background and objectives:**

Conduct disorders are associated with deficits in interpersonal behaviour. Both, callous-unemotional traits and social anxiety are often elevated in patients with conduct disorder and are associated with aggressive approach or disproportional avoidance. Previous studies have focused mainly on questionnaire reports of interpersonal behaviour, whereas direct explicit and implicit interpersonal behaviour in social contexts has not been considered sufficiently. Therefore, explicit and implicit interpersonal behaviour were investigated in children and adolescents with conduct disorder in the current study.

**Methods:**

Forty male adolescent inpatients with conduct disorder and 30 typically developing controls (*M*_*age*_ = 12.5, *SD* = 1.39) took part in a virtual reality task in which they approached virtual agemates, displaying different facial expressions under the pretext of a cover story while interpersonal distance and walking speed were assessed (indirect condition). In addition, they were asked to move to a comfortable distance for conversation toward the agent (direct condition). Callous-unemotional traits and social anxiety were assessed via questionnaires.

**Results:**

In the indirect condition, no differences between the groups emerged. In the direct condition, typically developing children adjusted their interpersonal distance to the respective expression that the virtual classmate displayed. They showed significantly greater interpersonal distances to angry classmates than to happy classmates. In contrast, conduct disorder patients’ interpersonal distance, did not differ between emotions. Interpersonal distance preferences were also associated with social anxiety and callous-unemotional traits.

**Conclusion:**

The findings suggest that conduct disorder patients fail to adjust their interpersonal behaviour to the facial expression of social interaction partners and that this is associated with social anxiety and callous-unemotional traits. A lack of adjustment to social cues might contribute to and maintain problems with peers in individuals with conduct disorder.

**Supplementary Information:**

The online version contains supplementary material available at 10.1186/s13034-024-00831-y.

## Introduction

Conduct disorder (CD) is among the most prevalent psychiatric disorders in childhood and adolescence, occurring in up to 8% of minors, depending on age and gender [1]. It is one of the most common reasons for mental health service usage (e.g., [2]). Across the lifespan, CD is associated with lower academic achievement and a greater risk for other mental disorders, criminality, and violence [3]. Meta-analyses show that treatments for CD only have small effects and emphasise the need for a better understanding of the underlying mechanisms and behavioural consequences of the disorder (e.g., [4]).

The core symptoms of CD relate to social functioning: Patients show aggressive, oppositional, and antisocial behaviour that ranges from arguing with authorities, deceitfulness, and irritating others to severe levels of violence, theft, rule-breaking or vandalism [5]. Affected children and adolescents have problems adhering to social rules and norms, resort to bullying, suffer from peer rejection, and struggle to maintain healthy relationships (e.g., [6]).

Within CD, these interpersonal deficits are typically associated with callous-unemotional (CU) traits. CU-traits are characterised by a lack of guilt and remorse, deficits in empathy, deficient emotionality, and disregard for one’s own performance [7]. A high level of CU-traits is associated with stronger and more persistent CD-symptoms such as deficits in social functioning: for example, CU-traits are linked to diminished empathy for victims of aggression and general affective empathy, reduced recognition of and response to fearful and sad facial and vocal expressions, less responsiveness to distressing stimuli, lack of avoidance of social threat, and increased reactive and proactive aggression [7, 8]. CU-traits are also linked to lower and less frequent experiences of fear as well as elevated levels of fearless behaviour [7]. Interestingly, although CD-patients exhibit elevated levels of CU-traits associated with fearlessness, they also tend to show elevated levels of anxiety (e.g., [9]), and CD and social anxiety (SA) disorder significantly co-occur [10].

SA (disorder) is characterized by high levels of fear in social situations and avoidance of those situations [11] and has, like CU, been linked to problems with interpersonal behaviour (IPB) and social functioning. For example, SA is linked to relational as well as reactive aggression [12], lower friendship quality, greater peer rejection and peer victimisation [13], and social performance deficits [14]. Although CU and SA show significant overlap in IPB-deficits, the pathways and underlying processes through which CU and SA influence IPB in CD are barely understood. There is evidence that interpersonal behaviour in general and that of CD children, in particular, might be associated with disrupted evaluation or recognition of social cues. For instance, studies have shown that CD is associated with facial emotion recognition deficits and that these deficits are associated with CU-traits and comorbid anxiety (e.g., [15, 16]). These emotion recognition deficits might be a proxy of interpersonal deficits in real social situations– in fact, Kohls and colleagues [17] linked emotion recognition deficits to physical and proactive aggression. Studies measuring IPB in social situations more directly via experimental behavioural tasks also yielded interesting results: Gaule and colleagues [18], for example, investigated social behaviour in a series of computer games. They found that adolescents with conduct problems (CP) had difficulties recognising trustworthy and untrustworthy social environments and that CU-levels influenced social cooperation. In a study by Derks and colleagues [8], CD-patients and TD-agemates performed a computerised approach-avoidance task (AAT) with photos of emotional faces (angry, happy, neutral). The results showed that CD was associated with a lack of automatic avoidance of angry faces and that increases in CU-traits predicted this lack of threat avoidance. Dapprich and colleagues’ [19] research, for instance, delivered comparable results in adults displaying psychopathic or CU-traits while adults with SA showed increased avoidance of angry faces (e.g., [20]). In an attempt to transfer the impulsive (lack of) avoidance to real-life social situations, Kroczek and colleagues [21] employed interpersonal distance (IPD) tasks to measure implicit and explicit social behaviour directly. In these tasks, participants are typically required to approach virtual human agents (active approach) or to let virtual agents approach them until a certain point of (dis)comfort (passive approach) is reached. This can be done directly by asking participants to establish a comfortable distance for conversation (preferred IPD) or indirectly by using a cover story (e.g., let participants recognize the virtual agent’s name on a name tag). For example, Welsch and colleagues [22] asked students to approach human agents displaying either angry or happy facial expressions in a virtual reality (VR) environment until a comfortable IPD had been reached. The authors found that self-reported psychopathic traits did not change mean IPD but rather its regulation– students with low psychopathic traits held a greater distance from angry than from happy agents, while students with elevated psychopathic traits adapted their IPD to a lesser extent to the agent’s expression. Further studies have established that in adults, psychopathic traits are associated with a preference for shorter IPDs [23], and a slower avoidance of anger [24]. The evidence regarding SA is mixed. While SA was associated with a generally greater IPD toward agents in two studies, approach speed was found to be faster in one of those [25] or slower in the other [26].

In sum, research shows that psychopathy and SA are associated with altered approach-avoidance reactions. While SA is associated with implicit (automatic/impulsive) and explicit (deliberate) avoidance, psychopathy seems to be characterised by a lack of implicit and explicit avoidance behaviours in experimental behaviour tasks. Children and adolescents diagnosed with CD also show deficits in interpersonal behaviour; however, social approach and avoidance behaviours have not yet been systematically investigated. Based on previous findings, it seems likely that children and adolescents with CD show alterations in social IPB and that these alterations are associated with the levels of anxiety and CU-traits. More insight into the underlying processes of CD are crucial for understanding pathways involved in dysregulated social behaviour and enhancing therapies accordingly. For example, von Borries and colleagues [27] found that adult individuals with psychopathy lacked avoidance of angry faces on an AAT and that this lack of avoidance was associated with instrumental aggression. Thus, approach-avoidance behaviour on experimental tasks might be associated with real-life aggressive behaviour. For this purpose, participants had to approach virtual agemates displaying different emotions to assess their eye colour (indirect condition) and to indicate their preferred interpersonal distance (direct condition). CU-traits and SA were assessed with questionnaires. We expected CD-patients to show a preference for a smaller IPD and a faster approach, measured by walking speed (WS), than controls. We expected IPD-preferences and WS to be especially different between groups when the virtual classmate had an angry expression. Furthermore, we hypothesised CU-traits to be linked to a faster approach and a shorter IPD and SA to a slower approach and a greater IPD.

## Methods

### Participants

Forty-six inpatients between 10 and 14 years of age diagnosed with CD based on the International Classification of Diseases (10th ed.; F90.1, F91.-, F92.-; [28]) were recruited from the LWL-University Hospital for Child and Adolescent Psychiatry Hamm in Germany. The exclusion criteria were an IQ < 85 or disorders that impaired the ability to participate in the study (e.g., acute psychosis, acute suicidality). Of the 46 participants, six had to be excluded from the analysis for various reasons: hospital discharge before conducting the VR-task (*n =* 1), turning 15 years old in between screening and testing (*n =* 1), lack of a CD-diagnosis in a clinical screening interview (*n =* 1), technical problems during the VR-task (*n =* 3), resulting in a CD-sample of *n* = 40.

In addition, 32 TD-children were recruited from high schools in the Hamm area. Of the 32 participants, one had to be excluded from the analysis due to an IQ < 85, and another due to technical problems with the VR-task, resulting in a TD-sample of *n* = 30. The total sample of the study comprised *n* = 70 participants (*M*_*age*_ = 12.5, *SD* = 1.39). The study was approved by the local medical-ethical committee (Ruhr University Bochum, Nr. 20-6861; 18th May 2020).

### Measures

#### CU-traits

 In light of studies pointing to informant discrepancies on internalising as well as externalising symptoms [29], especially in adolescence and for social avoidance [30], we chose to assess parent- as well as self-report on CU and SA. While there was a significant and positive correlation between parent- and self-report for CU-traits (Spearman’s *ρ* = 0.323, *p* =.009), no significant association was found for SA (Spearman’s *ρ* = 0.109, *p* =.388). Therefore, both reports were included in the analyses of the current paper

CU-traits were measured with the German parent- and self-report versions of the Inventory of Callous-Unemotional Traits (ICU; [31]). The ICU contains 24 items scored on a 4-point Likert scale ranging from 0 (not at all true) to 3 (definitely true). The items describe indifference in relation to one’s own performance and feelings of others, lack of guilt, empathy, and remorse, and reduction or absence of emotional expression. By adding up all items, a total score can be derived. In the current sample, reliability was excellent for the parent-reported total score (Cronbach’s *α* = 0.90) and good for the self-reported total score (Cronbach’s *α* = 0.81).

#### Social anxiety

SA was measured with the German parent- and self-report version of the Social Anxiety Scale for Children - Revised (SASC-R-D; [32]). The SASC-R-D consists of 18 items scored on a 5-point Likert scale, ranging from 1 (never) to 5 (always). The items describe fear of negative evaluation as well as social avoidance and distress in various social situations. By adding up all items, a total score can be calculated. In the current study, the reliability of the total score was excellent for parent-reports (Cronbach’s *α* = 0.91) and for self-reports (Cronbach’s *α* = 0.92).

#### Intelligence screening

IQ was measured with the short version of the Wechsler Nonverbal Scale of Abilities (WNV; [33]). The WNV is a nonverbal measure of ability for children and adolescents aged 8–21 years. The short version consists of the two subscales “Matrices” and “Spatial Span”, and a total IQ score can be derived.

#### Structured diagnostic interview

The Diagnostic Interview for Children and Youth for DSM-5 (Kinder-DIPS, DSM-5 version; [34]) is a structured clinical interview to assess common mental disorders in childhood and adolescence based on DSM-5 criteria. The DIPS was completed with participants’ legal guardians.

### General psychopathology

General psychopathology was measured with the Child Behaviour Checklist (CBCL/6-18R; [35]). The CBCL is a parent rating scale assessing behavioural problems, emotional problems, somatic complaints and social competences of children and adolescents with 113 items. A total score as well as scores for the subscales “internalising” and “externalising behaviour problems” can be derived. In the current study, Cronbach’s α = 0.94 for the total score, 0.86 for the internalising subscale, and 0.91 for the externalising subscale.

#### Visual analogue scales

Mood was rated on five 10 cm long visual analogue scales (VAS; e.g., [36]) ranging from 0 (not at all) to 100 (very) based on the five emotions (i.e., neutral, happy, angry, sad, anxious) used in the virtual IPD-task.

To control for the possible influence of the outbreak of the pandemic shortly before the beginning of the study, two VAS were used. Participants were asked to rate (a) “What do you think, how much does the COVID-19 pandemic influence your behaviour at the moment?” and (b) “What do you think, how much has the COVID-19 pandemic influenced your behaviour in the VR-task?” on a 10 cm sliding scale ranging from 0 (not at all) to 100 (very much).

#### Virtual interpersonal distance task

Automatic approach and avoidance tendencies were measured with a virtual reality (VR) task. For this task, a virtual environment resembling a school cafeteria was created (see Fig. S.1). Participants wore a stereoscopic head-mounted display that immersed them in the VR-scenario and signalled the participant’s physical position and orientation in the room to the computer (15 Hz) to adjust the displayed scene accordingly. Participants had the ability to walk around freely. The research lab had objects (tables and chairs) located at the same locations as in the VR-environment to increase the immersiveness of the virtual world. Immersiveness was further strengthened by typical cafeteria acoustics (e.g., voices, clattering crockery).

*General task setup.* During the task, the participant encountered a virtual agent in a school cafeteria. A cover story was used, which included the agent being a new classmate whom the participant would meet for the first time at the school cafeteria during a lunch break. On each trial, the participant stood with his back toward the agent behind a yellow starting line at 5.3 m away from the agent. The participant was instructed to turn to the agent after an acoustic signal (the agent said hi/hey/hello; prerecorded with real children’s voices). The participant was then supposed to walk toward the agent. The agent’s age and height were adapted to the participant’s age and height. The agent’s body was oriented toward the participant’s starting point and during the trial, his head moved to maintain an orientation toward the participant. After completion of the trial-specific task, the participant was instructed to return behind the yellow line. When the participant stood with his back to the agent behind the starting line again, the next trial with a new agent started. For the task, different virtual agents (see Fig. S.1) were created that appeared in a randomized order. Throughout all trials, the agents displayed a nonspecific greyish eye colour that changed gradually with the approach of the agent; the change started at 1.5 m away from the agent and was finalized at 1 m from the agent to ensure that participants walked close enough to the agent and paid attention to the facial region. The complete VR-task lasted for approximately 15–20 min. The minimal distance participants held to the agent (interpersonal distance, IPD) on each trial and the walking speed (WS) while approaching of the agent were the variables of interest recorded during the task.

*Practice trials.* The task started with three practice trials in which the participant encountered a virtual robot to become acquainted with the task setup and the VR-environment. The participant was instructed to recognize the robot’s eye-colour and walk back behind the yellow line after each eye-colour recognition.

*Indirect trials.* During these trials, virtual agents were boys introduced as new classmates. The task was the same as in the practice trials: participants were instructed to recognize the virtual classmate’s eye colour. The indirect phase consisted of 23 trials: (1) one practice trial with a virtual classmate maintaining a neutral emotional expression throughout the whole trial, (2) four trials with a neutral emotional expression, (3) two emotional practice trials with a task-irrelevant emotion (disgust), and (4) 16 emotional trials (4 emotions X 4 repetitions) with task-relevant emotional expressions (i.e., anger, anxiety, happiness, sadness) in a randomized order. The same emotion was never presented more than twice in a row. All emotional trials started with the agent displaying a neutral facial expression that gradually changed into a distinct emotional expression during approach. The change started at 4 m from the agent and was finalised within 2 s (or at latest 3 m) from the agent.

*Direct trials.* The direct phase consisted of four emotional trials in which different agents showed every task-relevant emotion once. Participants were instructed to approach the agent up to a distance where they found it comfortable to begin a conversation. Then they were supposed to greet the agent loudly, again imagining that they met a new classmate in their school’s cafeteria.

#### Emotion recognition

Explicit emotion recognition (ER) was tested with a computerized emotion recognition task (ERT) to check whether participants were able to identify the emotional expressions shown by the virtual agents in the VR-task. Participants were presented with screenshots of the full-blown emotional expressions (i.e., neutral, happy, angry, anxious, or sad) of the agents in the VR-task on a standard lab-computer. The face appeared in the middle of the computer screen and had to be categorised to the respective emotional label. Participants had to categorise 25 faces (5 expressions X 5 repetitions; see Figure S.2).

### Procedure

All information, questionnaires, and instructions were presented in German. Patients admitted to the hospital with a CD-diagnosis and their parents were contacted for the study. Healthy controls were recruited at local schools through information events, flyers, and posters in public places. Potential participants and their legal guardians were informed about the study verbally and in writing and were given the opportunity to ask questions. After informed consent was received, the Kinder-DIPS was conducted with legal guardians for screening purposes. For the control group, the entire Kinder-DIPS was administered to ensure that the child did not suffer from any clinically relevant mental disorders. For the patient group, the diagnosis of CD was verified by assessing the respective conduct disorder sections of the Kinder-DIPS. After that, legal guardians completed the parent-report questionnaires (ICU, SASC-R-D, CBCL).

Participants were tested in individual sessions at the clinic’s VR-lab. For patients, testing took place in the morning prior to taking medication to minimize drug effects. The WNV was conducted with participants if no prior IQ-measurement had taken place within the previous half year. After completing the self-report questionnaires (ICU, SASC-R-D)^1^ and mood-VAS, participants were asked to execute the VR-task. Testing ended with the ERT and the Covid-VAS. The participants were thanked for their participation and rewarded with sweets. Controls received an additional small compensation for coming to the clinic’s VR-laboratory. No debriefing took place to prevent other potential participants from knowing about the real study goal.

### Data preparation and statistical analysis

VR-trials in which the researcher observed irregular behaviour of the participant (e.g., turning around on the way to the agent, stopping halfway to pose a question, or changing position of the VR glasses) were excluded from the analysis (*n*_*ipd*_ = 60, *n*_*ws*_ = 70). Additionally, for the indirect phase, trials were excluded from the IPD analyses if the IPD was less than 20 cm from the agent because with an IPD below 20 cm, the participant is likely to have walked into the agent and therefore did not adhere to the task instructions. This resulted in the exclusion of 21 trials (1.5% of trials). One participant was excluded completely from the analysis of indirect trials as nearly half of his trials had to be deleted due to extremely low IPD-values. Missing values on questionnaires were analysed and found to be missing completely at random (Little’s MCAR test: *chi*² = 537.12, *p* =.104). Missing data were replaced with the individual mean value on the respective questionnaire’s subscale. The ER-task had very few missing responses (0.4% of trials) which were not replaced as the percentage of correct responses was not dependent on the number of times a specific emotion was rated.

IBM SPSS Statistics 29 was used for the data analysis. Descriptive statistics were calculated for the variables of interest. Differences between groups with regard to all descriptive measures, ER error rates, VAS, and demographics were assessed with two-tailed independent samples t-tests or chi-square tests.

VR-data from indirect trials were investigated with two Repeated-Measures Analysis of Variance (rmANOVA) with Group (CD versus TD) as a between-subjects factor, Expression (Neutral, Angry, Happy, Anxious, Sad) as a within-subjects factor, and either WS or IPD as the dependent variable. VR-data from direct trials were investigated with a rmANOVA with Group (CD versus TD) as a between-subjects factor, Emotion (Angry, Happy, Anxious, Sad) as a within-subjects factor, and IPD as the dependent variable.

To better understand which facet of CU/SA contributed to significant associations with IPB and to control for individual differences in ER and the impact of COVID-19, 2-step hierarchical regression analyses across the whole sample were calculated. In these, ER and COVID-VAS-scores were included as predictors in the first step, either parent- or self-reported ICU and SASC total-scores in the second step, and those IPBs for which a significant group difference was found in the previous rmANOVAS were included as the dependent variables. This time, all participants, independent of their group were analysed together since CU-traits and SA are transdiagnostic phenomena that occur in the general population independently of psychopathology (e.g., [41]).

Because of the unequal group sizes (40 vs. 30), the resulting statistical power for the rmANOVAS was computed using a conservative estimate of the sample size (two groups of 30). For the critical mixed-factors 2 × 5 interaction on indirect trials, the power to find it significant was 1-*ß* > 0.99 for a large interaction effect, 1-*ß* > 0.99 for a medium-sized effect, and 1-*ß* = 0.46 for a small effect (all with *p* =.05 and *r* =.50). For the critical mixed-factors 2 × 4 interaction on direct trials, the power to find it significant was 1-*ß* > 0.99 for a large interaction effect, 1-*ß* > 0.99 for a medium-sized effect, and 1-*ß* = 0.42 for a small effect (all with *p* =.05 and *r* =.50). For the regression analyses, the power was 1-*ß* > 0.99 to detect large predictor effects (*f*^*2*^ = 0.35), 1-*ß* = 0.93 for medium-sized effects (*f*^*2*^ = 0.15), and 1-*ß* = 0.31 for small effects (*f*^*2*^ = 0.02).

## Results

### Descriptive statistics

Descriptive statistics and group differences for control variables (i.e., emotion recognition, COVID-19 VAS, and mood VAS can be found in the supplement (Table S.1).

When comparing sample descriptives between the conduct disorder (CD) group and typically developing (TD) group (Table 1), it appeared that CD-patients, as expected, had significantly higher levels of callous unemotional (CU) traits, general, internalising, and externalising psychopathology. Interestingly, while social anxiety (SA) scores were also greater in the CD group, this effect reached only marginal significance.

**Table 1:** Descriptive statistics and sample differences for the CD- and TD-groups

	CD (*n* = 40)	TD (*n* = 30)	t-statistic	Cohen’s d
	M (SD)	M (SD)		
Age	12.45 (1.39)	12.47 (1.40)	− 0.051	− 0.012
IQ	96.45 (7.50)	104.13 (12.59)	− 2.971**	− 0.769
Medication *n (%)*	17 (42.5)			
No. of diagnoses	1.85 (0.84)			
Comorbidity *n (%)*	25 (62.5)			
ICU	35.56 (10.13)	17.7 (6.38)	8.79***	2.062
SASC	46.25 (12.27)	40.77 (9.77)	1.99^†^	0.488
CBCL	75.33 (9.14)	49.93 (6.18)	12.95**	3.200
INT	68.61 (7.69)	49.73 (7.32)	10.15**	2.509
EXT	75.78 (10.92)	48.37 (7.02)	12.32**	2.930

Group differences were tested with two-sided t-tests. CD = conduct disorder patients; TD = typically developing peers; ICU = parent-reported Inventory of Callous-Unemotional Traits total scores; SASC = parent-reported Social Anxiety Scale for Children total scores; CBCL = parent-reported Child Behaviour Checklist total scores, scores on the CBCL-internalising (INT) and -externalising (EXT) subscales

***p <.001,  **p <.01,  *p <.05, ^†^*p* <.10

### Approach-avoidance behaviour

Descriptives of interpersonal distance (IPD) and walking speed (WS) toward emotional virtual classmates were calculated for direct and indirect trials per group (Table S.2). Control analyses were performed to assess whether the respective agent, eye colour, or trial number had a significant influence on IPD and WS during indirect trials.^2^

### Walking speed

WS was only measured in *indirect trials.* Neither the main effect of group on WS, *F*(1,67) = 3.117, *p* =.082, *η²*_*ρ*_ = 0.044, nor the expected interaction effect of expression and group, *F*(2.97, 199.13) = 0.508, *p* =.676, *η²*_*ρ*_ = 0.008, were significant. A significant main effect of expression emerged, *F*(2.97, 199.13) = 11.745, *p* <.001, *η²*_*ρ*_ = 0.149. WS toward neutral agents was significantly slower than WS toward all other emotional agents (all *p*s *≤* 0.016). There were no significant differences between all other emotions concerning WS (all *p*s *≥* 0.109).

### Interpersonal distance

#### Indirect trials

The rmANOVA did not reveal the expected main effect of group, *F*(1,67) = 0.836, *p* =.364, *η²*_*ρ*_ = 0.012, or the interaction effect of expression and group, *F*(2.36, 157.87) = 0.524, *p* =.623, *η²*_*ρ*_ = 0.008, on IPD. Nevertheless, there was a significant main effect of expression, *F*(2.36, 157.87) = 3.204, *p* =.035, *η²*_*ρ*_ = 0.046. Only the difference between angry trials and sad trials, *M*_*diff*_ = 0.033 m, *SE* = 0.011, *p* =.031, as well as between angry trials and anxious trials, *M*_*diff*_ = 0.037 m, *SE* = 0.009, *p* =.002, reached significance.

#### Direct trials

**Fig. 1:**
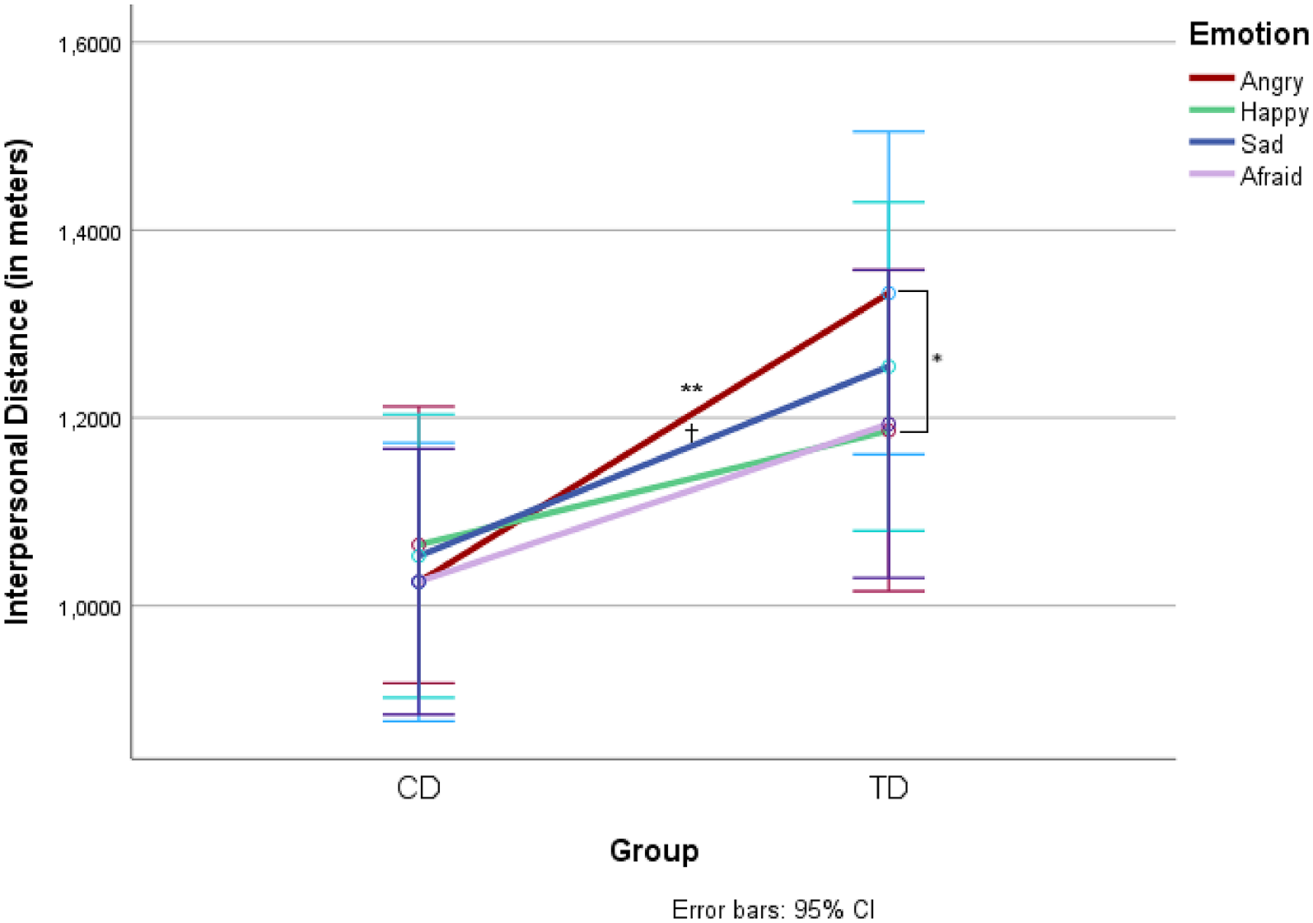
Explicit interpersonal distance during approach of virtual classmates. Mean IPD in meters (y-axis) between groups (x-axis; CD, TD) and emotions (angry, happy, sad, anxious) on the VR-task during direct trials.  **p  <.01,  *p  <.05,  ^†^p  <.10.

The rmANOVA revealed no significant main effect of group, *F*(1,66) = 3.582, *p* =.063, *η²*_*ρ*_ = 0.051, or emotion, *F*(2.66, 175.33) = 1.799, *p* =.156, *η²*_*ρ*_ = 0.027. The expected interaction effect of emotion and group was significant, *F*(2.66, 175.33) = 2.981, *p* =.039; *η²*_*ρ*_ = 0.043 (Fig. 1). CD-patients showed a significantly smaller IPD than TD-children on angry trials, *M*_*diff*_ = 0.308 m, *SE*_*diff*_ = 0.114, *p* =.009. No significant differences emerged between the groups on happy, sad, and anxious trials (*p*s *≥* 0.086). Within the CD-group, the IPD between emotions did not differ significantly (all *p*s > 0.999). Within the TD-group, participants showed a significantly greater IPD, *M*_*diff*_ = 0.146 m, *SE*_*diff*_ = 0.051, *p* =.034, toward angry agents than toward happy agents. No significant differences were found between the other emotions (all *p*s *≥* 0.128).

### Follow-up analyses: associations between approach-avoidance behaviour and participant characteristics

Two hierarchical multiple linear regression analyses (parent-report vs. self-report; Table 2) were performed to better understand the associations between participant characteristics (CU, SA) and IPD in angry trials, controlling for the ER of angry faces and the impact of COVID-19 on participants’ everyday lives (COVID).

**Fig. 2:**
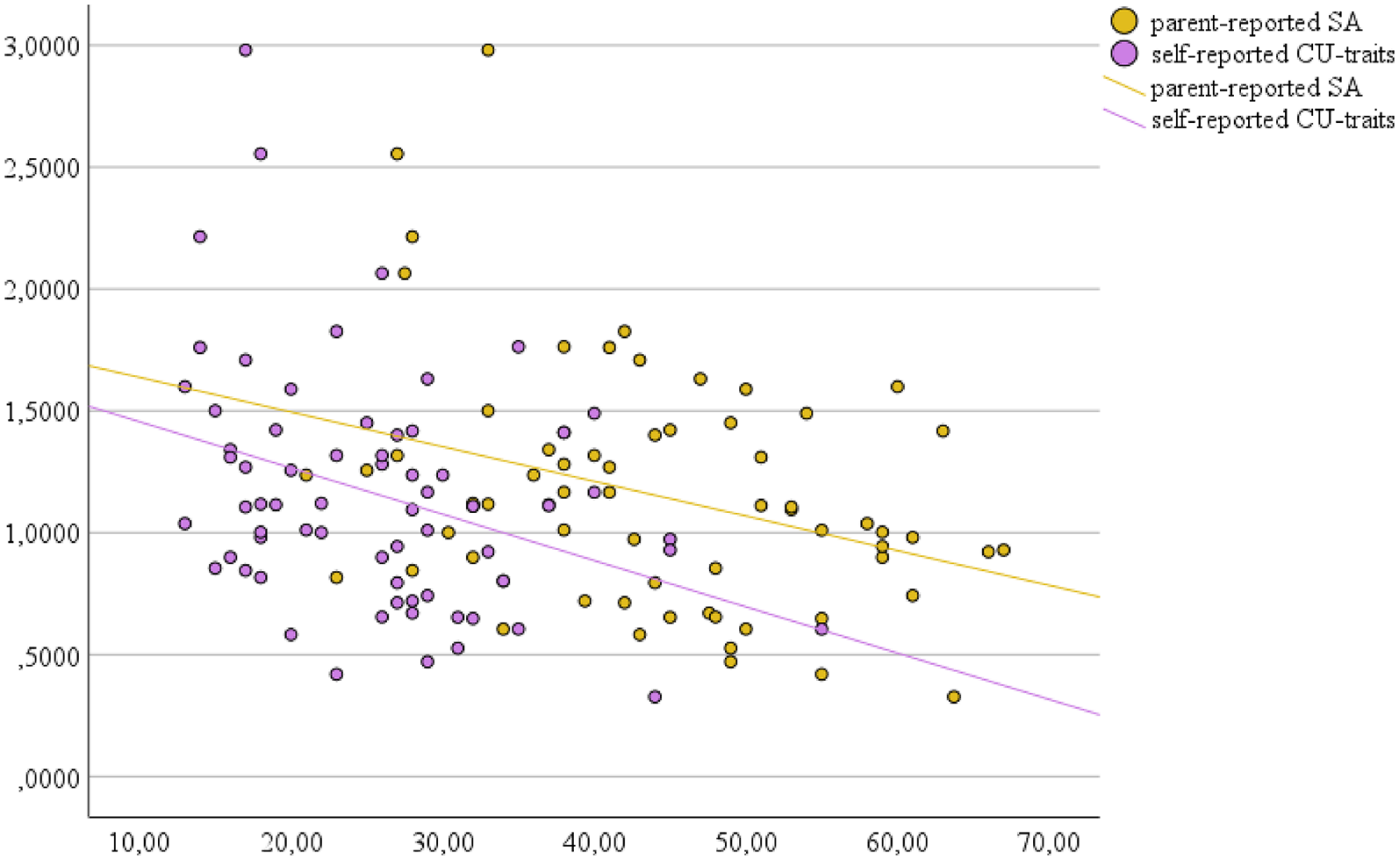
Prediction of explicit interpersonal distance to angry agemates from social anxiety and callous-unemotional traits. Mean preferred IPD in meters (y-axis) toward angry virtual classmates on the VR-task predicted from the parent-reported SASC-R-D total score (x-axis; yellow dots and line) or self-reported CU-traits (x-axis; purple dots and line) across both groups

While the first step of the *parent-report-based regression model* was nonsignificant, *F*(2, 66) = 0.632, *p* =.535, *R²* = 0.019, the second step reached significance, *F*(4, 66) = 3.025, *p* =.024, *R² =* 0.163. In the second step of the regression model, IPD toward angry classmates was significantly predicted by parent-reported SA. Higher SA predicted a preference for shorter IPDs (Fig. 2). A 1-point increase in SA-scores led to a decrease in preferred IPD of 1.3 cm.

While the first step of the *self-report-based regression model* was nonsignificant, *F*(2, 69) = 0.78, *p* =.459, *R²* = 0.023, the second step reached significance, *F*(4, 69) = 3.165, *p* =.019, *R² =* 0.163. In the second step of the regression model, IPD toward angry classmates was significantly predicted by self-reported CU-traits. Higher CU-traits predicted a preference for shorter IPDs (Fig. 2). A 1-point increase in CU-scores led to a decrease in preferred IPD of 1.9 cm.

**Table 2:** Hierarchical multiple regression analyses predicting approach-avoidance behaviour on the VR-task from CU-traits, social anxiety, emotion recognition, and COVID-19 impact

Variable	B	BCa 95% CI for B		SE B	β	*R*²	Δ *R*²
		LL	UL				
*Parent-report*							
Step 1						0.019	
Constant	1.240	0.163	2.376	0.529			
ER Angry	0.000	-0.010	0.011	0.005	0.008		
COVID	-0.023	-0.066	0.016	0.022	− 0.140		
Step 2							
Constant	2.067	0.674	3.570	0.656		0.163*	0.144**
ER Angry	-0.001	-0.011	0.008	0.005	− 0.023		
COVID	-0.001	-0.045	0.043	0.023	− 0.008		
CU	-0.009^†^	-0.018	-0.002	0.005	− 0.227		
SA	-0.013*	-0.024	-0.003	0.006	− 0.305		
*Self-report*							
Step 1						0.023	
Constant	1.160	0.007	2.387	0.541			
ER Angry	0.001	-0.009	0.012	0.005	0.031		
COVID	-0.024	-0.065	0.014	0.021	− 0.152		
Step 2							
Constant	1.689	0.469	3.036	0.666		0.136*	0.140**
ER Angry	0.001	-0.009	0.011	0.005	0.023		
COVID	− 0.027	-0.065	0.006	0.020	− 0.168		
CU	− 0.019**	-0.033	-0.008	0.006	− 0.373		
SA	0.000	-0.008	0.011	0.005	0.013		

CI = confidence interval; *LL* = lower limit; *UL* = upper limit; CU = Inventory of Callous-Unemotional Traits (ICU) total score; SA = Social Anxiety Scale for Children (SASC-R-D) total score; ER Angry = emotion recognition of angry faces on the Emotion Recognition Task; COVID = participants’ VAS-rating of the impact of COVID-19 on their real life. The 95% bias corrected and accelerated confidence intervals and standard errors were based on 1000 bootstrap samples

***p <.001, **p <.01, *p <.05, ^†^*p* <.10

## Discussion

The aim of the present study was to assess interpersonal behaviour (IPB) tendencies in children and adolescents with conduct disorder (CD) and their associations with callous-unemotional (CU) traits and social anxiety (SA). We hypothesized that, in contrast to typically developing (TD) agemates, CD-patients would show a preference for shorter interpersonal distances (IPDs), and faster walking speed (WS), especially toward angry virtual classmates, and that these behavioural tendencies would be related to CU-traits and SA.

### CD and implicit social behaviour

We did not find a difference between CD- and TD-participants with regard to IPB in indirect trials: the groups did not differ in their WS or IPD toward agemates, irrespective of emotion. However, a significant main effect of emotion was found. Across groups, participants displayed a slower WS toward neutral agents than toward other emotions. This is likely to be an artefact of the task set-up: neutral agents were not part of the randomisation of emotions but were always presented as the first five trials. After these first five neutral trials, the other emotions occurred in a randomised order. It is possible that participants habituated to the repetitive nature of this part of the trials. It could also be that, after the trials with the robots and the first ‘real’ agents, participants had embodied/automatized the task instructions sufficiently to securely fulfil them later. The following, randomized trials would then not be affected by further habituation. Regarding IPD, participants held a closer distance to angry agents than to sad and anxious agents. Thus, the results concerning implicit social behaviour did not support our hypotheses which was primarily based on one previous study that assessed indirect approach-avoidance behaviour in CD-patients: Derks and colleagues [8] reported that, while TD-children showed an avoidance bias toward angry facial stimuli on an implicit approach-avoidance task (AAT), CD-patients lacked that avoidance and even showed an approach of angry faces. An explanation for the difference in findings might be the difference in measurement methods: the AAT-study might have been more sensitive for capturing implicit behaviour than the VR-paradigm. Participants on the AAT are instructed to push or pull stimuli as fast as possible by means of a computer joystick. These hand movements might be more easily adjusted to stimuli in the environment than the whole-body movement on the VR-task. The AAT might also elicit the feeling of being approached, while the VR-task is characterised by having control when approaching someone and might, therefore, be perceived as less threatening. Moreover, the AAT included a zoom function: when pulling pictures with the joystick, they increased in size. This facilitated emotion recognition (ER) for participants. While the approach of the agents naturally resulted in the agents (and their faces) becoming larger, the VR-paradigm did not include a specific zoom on the facial region. However, as IPD varied as a function of emotions on implicit trials, it is unlikely that participants did not perceive emotions correctly in the current study. Counterintuitively, participants even held the closest distance to angry faces. It might be that adolescents found these faces especially interesting and therefore approached them more closely. Another explanation might be the difference between indirect and direct approach-avoidance paradigms. Welsch and colleagues [22] compared the results of direct and indirect AAT-conditions in a student sample. Their results match ours: while happy faces were approached and angry faces were avoided in the direct AAT-condition, neither type of face elicited a facilitated approach or avoidance response in the indirect AAT-condition. There is generally a lack of studies investigating the more ecologically valid VR-IPD-paradigms and those studies that do exist primarily include adult samples. It might be that developmental samples generally show greater variance in IPD than adults. When comparing the variance in IPD between our sample and the adult sample used in Ruggiero and colleague’s study [42], our sample showed slightly larger *SD*s with regard to angry faces and substantially larger *SD*s with regard to happy faces. Therefore, especially developmental studies might need larger sample sizes to detect effects.

### CD and explicit social behaviour

In line with our hypothesis, the results revealed a difference between CD- and TD-children in angry trials, with CD-children holding a shorter distance to angry classmates than TD-children. However, rather than reflecting the approach of angry agemates in the CD-group, the interaction is driven by a general lack of approach-avoidance modulation in the CD-group and a significant avoidance of angry faces compared to happy faces in the TD-group. These results are in line with previous AAT- (e.g., [19, 27]) and IPD-studies (e.g., [22, 24]) in adults. The behaviour of the TD-group matches the pattern typically found in healthy samples: happy faces elicit approach while angry faces elicit avoidance and is a preliminary extension of these results to adolescent samples. The results of the CD-sample are in accord with other findings that relate CD to social interaction- and relationship problems (e.g., [6]). The failure of CD-patients to adjust their behaviour to their (virtual) social interaction partner’s emotions might be a marker of lessened attention to social cues during interactions. Especially when confronted with angry interaction partners, children with CD seemed to ignore the implicit message to stay away and maintained a greater distance. The shorter distance they kept might be perceived as an intrusion into the other’s personal space and could be perceived as threatening or make others feel uncomfortable. This, in turn, might contribute to the peer rejection often experienced by children with CD, as reported in the literature [6]. Another reason underlying the lack of avoidance of anger might be that CD-patients are at greater risk of living with parents using a harsh and punishing parenting style (e.g., [5]). This experience of aggression within the patients’ family and everyday life might have influenced IPB. For example, the frequent experience of angry interaction partners might have led to a habituation, resulting in the lack of avoidance found in the VR task. However, the difference between implicit and explicit findings, deem this explanation less likely. More studies with bigger sample sizes are needed to replicate our results and relate them to the home-situation of the young patients.

Moreover, the results of the CD-sample in the current study resemble those of adults with psychopathic traits. Welsch and colleagues [22, 24], and Dapprich and colleagues [19] studied the association between psychopathy and approach-avoidance patterns in healthy adults: both research groups found that higher levels of psychopathy correspond to a diminished avoidance reaction toward angry faces. Von Borries and colleagues [27] found the same effect in a clinical sample of adults diagnosed with psychopathy. These results are especially interesting in the light of the developmental pathway of a subgroup of CD-patients from CD in childhood and adolescence into psychopathy in adulthood [7]. This subgroup is primarily characterized by a high level of CU-traits and a low level of anxiety [7]. Therefore, we explored whether the lack of avoidance of angry faces was associated with CU-traits and SA in our sample.

### Participant characteristics and IPD

The follow-up regression analyses yielded an interesting pattern of results: As expected, higher levels of *self-reported* CU-traits corresponded to shorter IPD-preferences, thus less avoidance of angry faces. This matches studies showing that CU-traits are associated with fearless behaviour and are negatively related to general affective perspective-taking and empathy, feelings of fear and empathy for victims of aggression [7]. Moreover, CU-traits are associated with fewer concerns about the consequences of aggressive behaviour, such as punishment, victim suffering, and remorse, and with positive beliefs that aggressive behaviour is acceptable in social situations [7]. Shorter IPDs might then reflect carelessness about other people’s discomfort and personal boundaries. Biologically, CU-traits are associated with less responsivity to distress cues and lower skin conductance levels in response to aversive stimuli [7]. A lack of arousal in response to a socially distressing stimulus (i.e., an angry virtual classmate) might also result in maintaining a smaller distance. It remains unclear why parent-reported CU-traits were not significantly predictive of IPD. One reason might be that CU-traits are personality traits rather than observable and direct behaviour and that parents were less reliable in rating CU-traits. Studies investigating informant discrepancies show that correlations between informants’ ratings are lower in adolescence than childhood and for internalising than externalising problems [43]. In the current study, parent- and self-reports correlated significantly but only moderately– this shows quite some disagreement between parents and children. Moreover, in the parent-report-based regression, CU-traits showed a trend toward significance. Therefore, the difference in results of different informants might also be an issue of statistical power, and the results of the current study should be interpreted with caution.

Regarding the association between IPD and *parent-reported* SA, the direction of effect was opposed to our hypothesis: a higher level of SA corresponded to a shorter IPD-preference. This finding does not align with results from adult samples in which SA was associated with larger IPDs [25, 26] and more substantial avoidance of emotional faces on AATs [44]. An explanation could be that in children and adolescents, confrontation with an angry virtual agemate might elicit a stronger negative affect than in adults, especially in children with a high SA-level. The literature points to a link between negative affect and reactive aggression (e.g., [45]) as well as between SA and reactive aggression [12]. Consequently, a shorter IPD toward angry agents in higher SA participants might signify a measure of reactive aggression: children with higher levels of SA might be more prone to negative arousal following the approach of the angry classmate and might overregulate this negative arousal by a closer approach, signalling a form of reactive behaviour perceived as aggressive by the interaction partner. Dixon and colleagues [46] reported that in adults, emotion-driven impulse control difficulties mediated the path from SA symptoms to aggression. Therefore, participants with higher degrees of SA in our sample might have shown more approach toward agents due to emotion-driven impulsivity in angry trials. More research is needed to test these associations in developmental samples and to see whether the association holds in a more extensive and more diverse sample. Yet, it remains unclear, why self-reported SA did not predict IPD-preferences. With regard to SA, parent- and child-ratings did not correlate. Studies show that social desirability strongly influences children’s ratings of social avoidance, a facet of social anxiety: greater social desirability was negatively related to ratings of social avoidance in children [30]. Moreover, in a clinical sample, adolescents reported significantly less SA than their parents, and self-reported SA had a low correspondence to objective psychophysiological measures of anxiety, indicating a greater validity of parent reports for this age and symptom dimension [29]. In the current study, the sample size might have been too small to discover less pronounced effects.

### Transferability to the real world

VR-paradigms are thought to have high ecological validity and studies show comparability between virtual- and real-world IPD-behaviours (e.g., [47]) not only in assessments but also with regard to therapeutic interventions (e.g., [48]). With regard to improving psychotherapeutic interventions, which show regrettably small effect sizes in patients with CD [4], our results might give a first hint to incorporating IPB training into CD-treatment. Training to pay more attention to other people’s social signals, such as facial expressions, and adjusting one’s own distance accordingly might reduce peer rejection and social conflicts in CD-patients and foster healthy social development. This is especially relevant for patients with high levels of CU-traits. More specifically, social behaviour could be trained with a VR-task that incorporates the findings of the current study. Participants could approach agemates in this task and could be encouraged to adapt their IPD to the social signals of the interaction partner. Here, patients should gain rewards when establishing a nuanced and appropriate distance to respective emotions rather than punishments for incorrect behaviour (e.g., [5]). The task should also take findings regarding ER deficits into account and train attention to the eye region during the task (e.g., [49, 50]). Interestingly, two VR-trainings tackling aggressive behaviour have been developed and tested in adults in forensic settings (i.e., Virtual Reality Aggression Prevention Therapy (VRAPT; [51]), Virtual Reality Game for Aggressive Impulse Management (VR-GAIME; [52]). However, both trainings did not yield long-lasting results, which emphasizes the need for early interventions. Moreover, next to VR applications, psychotherapists could incorporate psychoeducation about these behavioural deficits for parents and their children. Appropriate reactions to the emotions of social interaction partners could be trained using role plays in the sessions. It would be interesting to include parents in these role plays because evidence points to a heritability of CU-traits (and possibly their behavioural proxies) and parents are important role models for behaviour.

### Limitations

The present study has several strengths and limitations. A strength of the study is the ecologically valid VR-task. A further strength is that IPB was measured experimentally and objectively and not with questionnaires which are usually prone to response biases and social desirability (e.g., [30]). For participant characteristics, self- *and* parent-reported data were obtained.

A limitation of the study is the relatively small number of participants that took part in the study, especially in the TD-group. Power analyses revealed that power should have been sufficient to detect medium-large but not small effects. This could explain the lack of group differences in the indirect condition of the VR-task. The conclusion is in line with a meta-analysis on approach-avoidance behaviour tasks and affect [53]. The researchers concluded that explicit tasks consistently yield larger effect sizes while tasks that implicit tasks, which do not require conscious appraisals of the affective valence of stimuli, often yield small effects. Due to the small sample size, the relatively high number of analyses, the inconsistency of results between parent- and self-report, and the inconsistency between explicit and implicit measures, the results of the current study should be regarded as preliminary evidence and interpreted with caution.

The current study also recruited only male participants because CD is more often diagnosed in boys than in girls [5]. Future studies should test whether the results also hold for female samples. Moreover, shortly after the beginning of the study, the COVID-19 pandemic broke out. It remains unclear to what extend the pandemic influenced IPB in our study. Some studies investigating the effect of COVID-19 on IPD have shown that automatic approach-avoidance behaviour seems robust against pandemic influences (e.g., [54, 55]). However, no studies have investigated changes in IPD in developmental samples following COVID-19, yet. In the current study, the groups differed in their estimation of the impact of COVID-19 on their real-life behaviour with CD-patients perceiving a greater impact than controls. This might be due to stronger restrictions during the pandemic for patients in the psychiatric ward than for healthy controls. In contrast, there was no difference between groups in their low estimation of the impact of COVID-19 on their VR-behaviour. In the regression analysis, COVID-19 (impact on real life) had no association with IPD so that at least the perception of COVID-related impact on real-life was not linked to IPD-behaviour in VR.

## Conclusion

In sum, taking the limitations into account, our results show that children and adolescents with and without CD differ in their explicit IPB. Compared with controls, CD-patients maintained a shorter distance to virtual classmates when they showed an angry facial expression. This effect is largely explained by the fact that CD-participants failed to adjust their distance to the emotional expression of the virtual classmate, while TD-participants maintained a greater distance from angry than from happy agemates. Explicit IPB is associated with self-reported CU-traits and parent-reported SA. Higher levels of both characteristics are associated with a preference for shorter distances. Alterations in subtle approach-avoidance behaviour might contribute to IPB-problems in CD and this might be fostered by CU and SA. Follow-up studies should replicate the results in larger samples including female participants. Possible underlying mechanisms of aberrant social behaviour and its relationships with anxiety and CU-traits should be further investigated to eventually improve the intersocial behaviours of children and adolescents with conduct disorder effectively.

## Supplementary Information

Additional file1 (DOCX 971 kb)

## Data Availability

The anonymized data can be requested from the first author upon reasonable request.

## Abbreviations

CD: Conduct disorder
CU-traits: Callous-unemotional traits
SA: Social anxiety
IPB: Interpersonal behaviour
CP: Conduct problems
TD: Typically developing
AAT: Approach-avoidance task
IPD: Interpersonal distance
VR: Virtual reality
WS: Walking speed
ICU: Inventory of callous-unemotional traits
SASC-R-D: Social anxiety scale for children– revised - Deutsch
WNV: Wechsler nonverbal scale of abilities
Kinder-DIPS: Diagnostic interview for children and youth for DSM-5
CBCL: Child behaviour checklist
VAS: Visual analogue scale
ER: Emotion recognition
ERT: Emotion recognition task
rmANOVA: Repeated-measures analysis of variance

## Footnotes

1 CD-patients also received additional self-report questionnaires (Ambiguous Social Scenario Task (ASST) [37], Test of Self-Conscious Affect [38], Compass of Shame Scale [39], Difficulties in Emotion Regulation Scale [40]), and TD-patients the ASST; however, these have no further relevance for the current paper.

2 No significant influences emerged for IPD and no significant influence of agent and eye-colour emerged for WS (all ps > 0.05). Across all 20 indirect trials, trial number had a significant effect (*p* <.001), which disappeared when including only the 16 randomized emotional trials (*p* =.216).

## Code availability

The software used is referenced in the text.
