# Supplementary material for: I can’t feel your face: callous-unemotional traits, social anxiety, and approach-avoidance behaviour in conduct disorder
Source: Child Adolesc Psychiatry Ment Health. 2024 Nov 27;18:153. doi: 10.1186/s13034-024-00831-y (PMC11603866; doi:10.1186/s13034-024-00831-y)
Supplement: Supplementary file 1 — Additional file1 (DOCX 971 kb) [file 13034_2024_831_MOESM1_ESM.docx]

**Supplementary Material**

**Figure S.1**

*
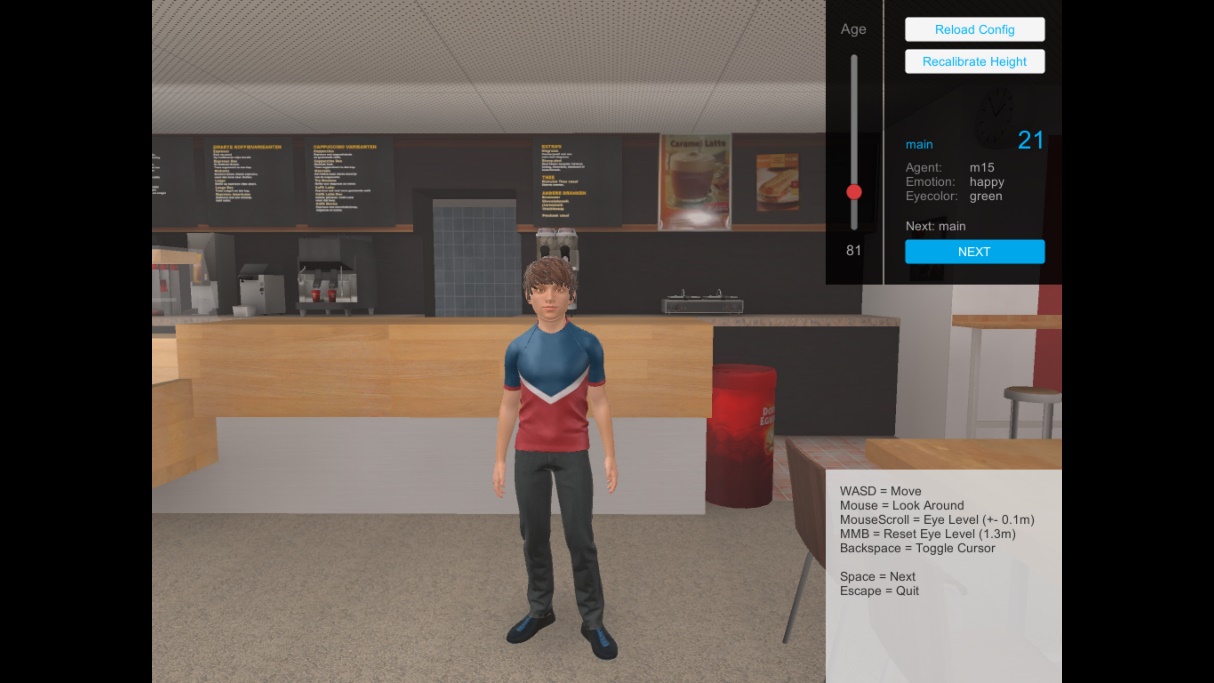

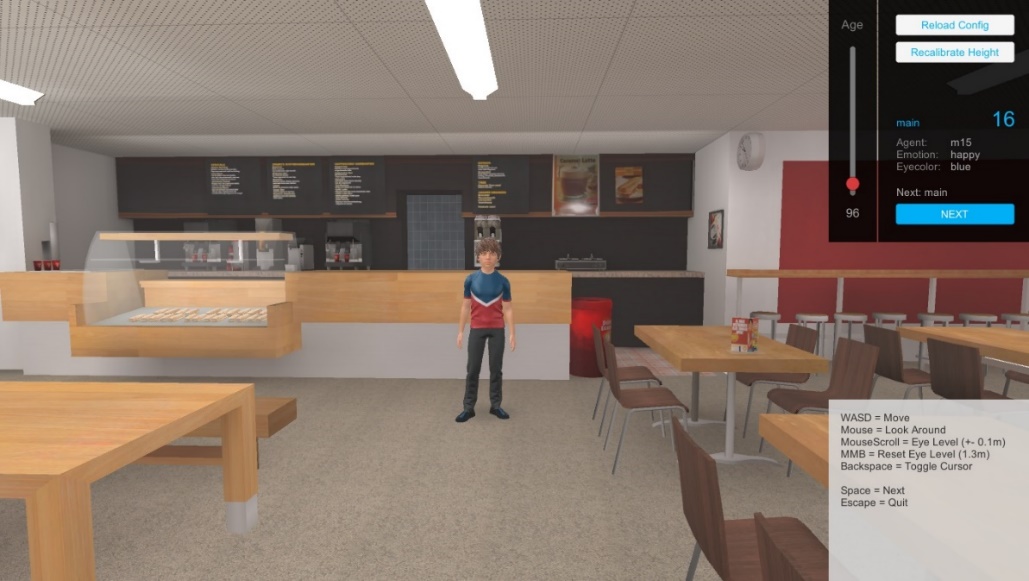
Example of a Virtual Classmate in the Virtual School Cafeteria*

**Figure S.2**

*Example Stimuli of the Virtual Reality- and Emotion Recognition Task*


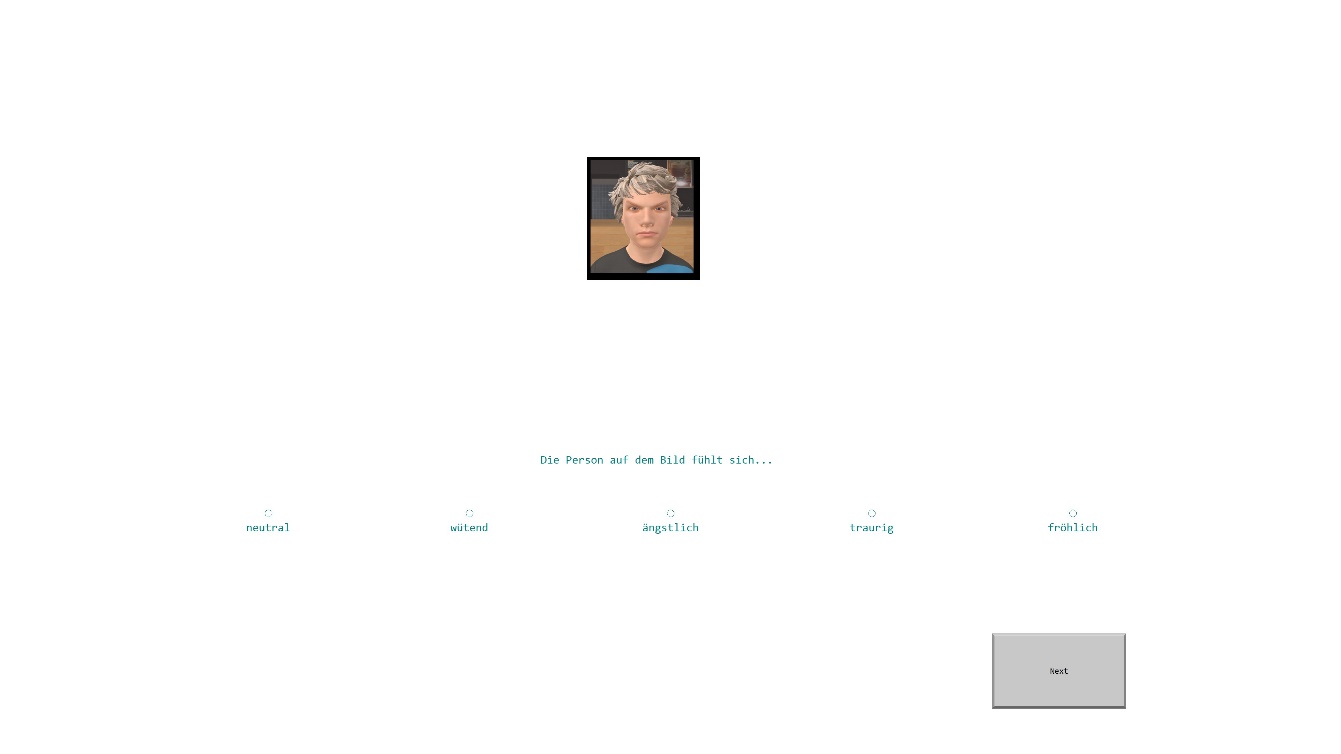

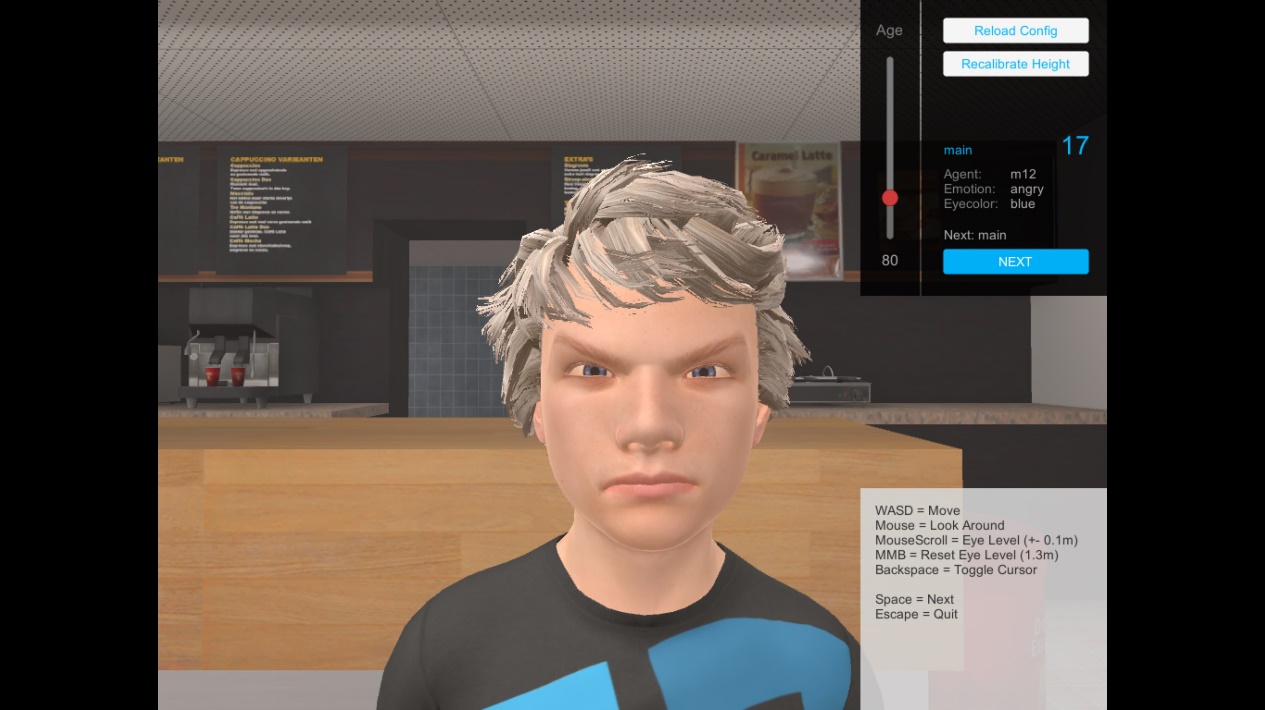

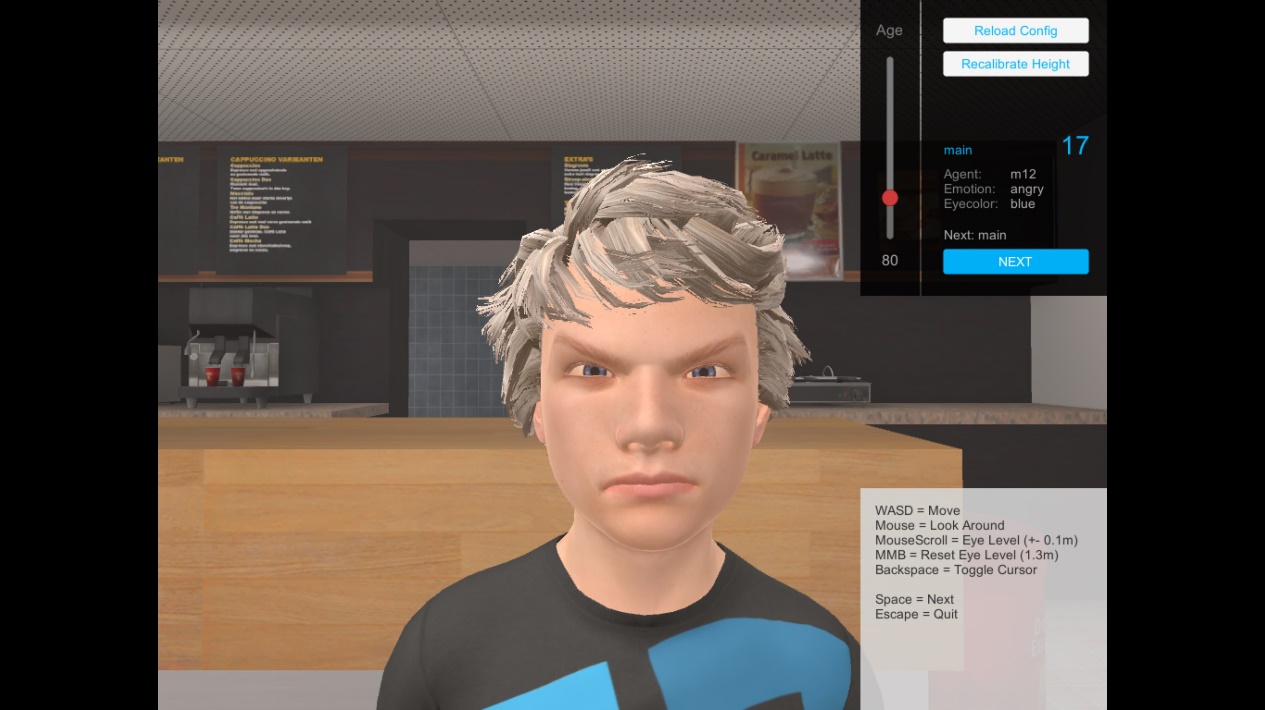


*Note:* Screenshot of the emotional face in the VR-task on the left panel. ERT-trial for rating this emotional expression on the right panel.

**Table S.1**

*Descriptives and Group Differences in Emotion Recognition and Visual Analogue Scales*

|  | CD (*n* = 40) | TD (*n* = 30) |  |  |
| --- | --- | --- | --- | --- |
|  | *M (SD)* | *M (SD)* | *t-statistic* | *Cohen’s d* |
| ERT |  |  |  |  |
| Neutral | 84.00 (27.25) | 93.33 (19.18) | -1.681^†^ | -0.387 |
| Angry | 88.00 (11.81) | 92.67 (9.80) | -1.757^†^ | -0.424 |
| Happy | 93.50 (13.88) | 96.67 (9.22) | -1.145 | -0.261 |
| Sad | 86.25 (19.83) | 92.67 (15.30) | -1.528 | -0.356 |
| Anxious | 55.38 (31.45) | 76.67 (26.82) | -2.982^**^ | -0.720 |
| Total | 81.43 (11.92) | 90.40 (9.43) | -3.400^***^ | -0.821 |
| COVID-19 |  |  |  |  |
| Real-life | 63.07 (30.22) | 38.22 (24.51) | 3.368^***^ | 0.889 |
| VR | 36.44 (29.60) | 26.81 (23.26) | 1.473 | 0.356 |
| Mood |  |  |  |  |
| Neutral | 46.18 (30.05) | 71.27 (27.18) | -3.563^***^ | -0.870 |
| Angry | 4.97 (10.96) | 9.73 (15.02) | -1.522 | -0.370 |
| Happy | 71.24 (29.56) | 65.17 (15.53) | 1.101 | 0.248 |
| Sad | 15.97 (26.76) | 12.33 (15.59) | 0.708 | 0.161 |
| Anxious | 6.65 (12.39) | 7.20 (17.71) | -0.153 | -0.037 |

*Note.* Mean percentage and standard deviation of correct responses on the computerized emotion recognition task (ERT) per emotion, self-reported ratings of COVID-influence on behaviour in real-life and virtual reality (VR), and self-reported mood. CD = conduct disorder patients; TD = typically developing peers.

^***^ *p* < .001 ^**^ *p* < .01 ^*^ *p* < .05 ^†^ p < .10

**Table S.2**

*Means and standard deviations of approach-avoidance behaviour in the VR-task.*

|  | CD | | TD | | General | |
| --- | --- | --- | --- | --- | --- | --- |
|  | Indirect | Direct | Indirect | Direct | Indirect | Direct |
|  | *M (SD)* | *M (SD)* | *M (SD)* | *M (SD)* | *M (SD)* | *M (SD)* |
| IPD | *n = 39* | *n = 39* | *n = 30* | *n = 29* | *n = 69* | *n = 68* |
| Neutral | 0.630 (.239) | - | 0.656 (.222) | - | 0.642 (.231) | - |
| Angry | 0.618 (.226) | 1.025 (.436) | 0.666 (.210) | 1.333 (.499) | 0.639 (.219) | 1.156 (.485) |
| Happy | 0.644 (.242) | 1.065 (.477) | 0.690 (.218) | 1.186 (.438) | 0.664 (.231) | 1.117 (.461) |
| Sad | 0.646 (.235) | 1.053 (.486) | 0.704 (.203) | 1.255 (.452) | 0.671 (.222) | 1.139 (.479) |
| Anxious | 0.648 (.246) | 1.026 (.428) | 0.710 (.218) | 1.193 (.461) | 0.675 (.235) | 1.097 (.447) |
| Total | 0.638 (.229) | 1.042 (.430) | 0.686 (.198) | 1.242 (.431) | 0.659 (.216) | 1.127 (.439) |
| WS | *n = 39* |  | *n = 30* |  | *n = 69* |  |
| Neutral | 0.822 (.116) | - | 0.873 (.150) | - | 0.845 (.133) | - |
| Angry | 0.859 (.133) | - | 0.914 (.136) | - | 0.883 (.136) | - |
| Happy | 0.876 (.109) | - | 0.933 (.157) | - | 0.901 (.134) | - |
| Sad | 0.866 (.103) | - | 0.920 (.122) | - | 0.890 (.114) | - |
| Anxious | 0.881 (.112) | - | 0.915 (.126) | - | 0.896 (.119) | - |
| Total | 0.861 (.107) | - | 0.912 (.127) | - | 0.883 (.118) | - |

*Note.* Means and standard deviations of interpersonal distance (IPD) in m and of walking speed (WS) in m/sec for the respective emotions and conditions on the virtual reality (VR) task. CD = conduct disorder patients; TD = typically developing peers.
